# 5 Hz repetitive transcranial magnetic stimulation over the ipsilesional sensory cortex enhances motor learning after stroke

**DOI:** 10.3389/fnhum.2014.00143

**Published:** 2014-03-21

**Authors:** Sonia M. Brodie, Sean Meehan, Michael R. Borich, Lara A. Boyd

**Affiliations:** ^1^Department of Physical Therapy, Faculty of Medicine, University of British ColumbiaVancouver, BC, Canada; ^2^School of Kinesiology, University of MichiganAnn Arbor, MI, USA

**Keywords:** repetitive transcranial magnetic stimulation, stroke, hemiparesis, primary sensory cortex, upper extremity, motor learning

## Abstract

Sensory feedback is critical for motor learning, and thus to neurorehabilitation after stroke. Whether enhancing sensory feedback by applying excitatory repetitive transcranial magnetic stimulation (rTMS) over the ipsilesional primary sensory cortex (IL-S1) might enhance motor learning in chronic stroke has yet to be investigated. The present study investigated the effects of 5 Hz rTMS over IL-S1 paired with skilled motor practice on motor learning, hemiparetic cutaneous somatosensation, and motor function. Individuals with unilateral chronic stroke were pseudo-randomly divided into either Active or Sham 5 Hz rTMS groups (*n* = 11/group). Following stimulation, both groups practiced a Serial Tracking Task (STT) with the hemiparetic arm; this was repeated for 5 days. Performance on the STT was quantified by response time, peak velocity, and cumulative distance tracked at baseline, during the 5 days of practice, and at a no-rTMS retention test. Cutaneous somatosensation was measured using two-point discrimination. Standardized sensorimotor tests were performed to assess whether the effects might generalize to impact hemiparetic arm function. The active 5 Hz rTMS + training group demonstrated significantly greater improvements in STT performance {response time [*F*_(1, 286.04)_ = 13.016, *p* < 0.0005], peak velocity [*F*_(1, 285.95)_ = 4.111, *p* = 0.044], and cumulative distance [*F*_(1, 285.92)_ = 4.076, *p* = 0.044]} and cutaneous somatosensation [*F*_(1, 21.15)_ = 8.793, *p* = 0.007] across all sessions compared to the sham rTMS + training group. Measures of upper extremity motor function were not significantly different for either group. Our preliminary results suggest that, when paired with motor practice, 5 Hz rTMS over IL-S1 enhances motor learning related change in individuals with chronic stroke, potentially as a consequence of improved cutaneous somatosensation, however no improvement in general upper extremity function was observed.

## Introduction

Motor recovery typically plateaus by 6 months after stroke (Hendricks et al., 2002), leaving 55–75% of individuals with chronic functional impairments of the hemiparetic arm (Gresham et al., 1995). Despite the neurological deficits after stroke, the capacity for motor learning persists (Boyd et al., 2009; Vidoni and Boyd, 2009; Meehan et al., 2011a). This has led to an interest in adjunct interventions to positively augment motor learning and further enhance functional recovery in chronic stroke.

Repetitive transcranial magnetic stimulation (rTMS)^1^ is a non-invasive technique used to modulate local cortical excitability in a frequency-dependent manner (Maeda et al., 2000), for a period of time that outlasts the duration of stimulation (Chen et al., 2003). Immediately following stimulation, the aftereffects may be capitalized on by pairing it with skilled motor practice to promote use-dependent neuroplastic change (Cohen et al., 1998). As such, rTMS is a promising adjunct therapy for enhancing the sensorimotor benefits of motor skill practice. Past work has primarily considered the application of rTMS over the primary motor cortex (M1) in individuals with stroke. However, to date findings have been inconclusive, both when rTMS is delivered in isolation (Boggio et al., 2006; Fregni et al., 2006; Carey et al., 2010), and when it is paired with rehabilitation (Seniow et al., 2012; Talelli et al., 2012). Inconsistent results may stem from a number of factors, including non-standardized stimulation location within and across experimental sessions, a failure to pair rTMS with a well-controlled motor learning task, and an exclusive focus on the effects of rTMS on the descending motor system.

Though often overlooked, the ascending somatosensory system also plays a crucial role in the acquisition of new motor skills (Debas et al., 2010). Early animal studies demonstrated that disrupting somatosensory feedback by selectively ablating the primary sensory cortex (S1) prevents motor learning (Sakamoto et al., 1989; Pavlides et al., 1993). Similarly in humans, we have observed that disrupting somatosensation by applying inhibitory 1 Hz rTMS over S1 in healthy individuals prior to skilled motor practice decreases motor skill acquisition (Vidoni et al., 2010). Further, we have shown that greater proprioceptive deficit predicts less motor learning related change after stroke (Vidoni and Boyd, 2009). On the other hand, stimulation of the somatosensory system may be used to enhance motor learning. Electrophysiological studies using *in vivo* animal models have demonstrated that long-term potentiation can be induced in M1 pyramidal neurons using tetanic stimulation of S1, via reciprocal cortico-cortical afferents (Sakamoto et al., 1987; Iriki et al., 1991). In humans, peripheral somatosensory stimulation has been shown to induce cortical reorganization of M1(Hamdy et al., 1998), and when paired with motor practice, to enhance motor learning in individuals with chronic stroke (Celnik et al., 2007).

These findings have led to the hypothesis that modulating the excitability of the somatosensory cortex may influence motor learning. More specifically, that increasing the excitability of S1 prior to motor practice may potentiate the formation and/or strengthening of sensorimotor connections critical for the development of lasting changes in motor performance. The ability to directly stimulate S1 in humans using rTMS, however, has not been widely explored. High frequency (5 Hz) rTMS applied over S1 in healthy individuals induces sustained increases in cortical excitability as measured by sensory evoked potentials (Ragert et al., 2004). In addition, preliminary results from our group suggest that when paired with skilled motor practice, continuous theta-burst stimulation (cTBS), an inhibitory variant of rTMS, applied over *contralesional* S1 (CL-S1) enhances aspects of motor learning in individuals with chronic stroke (Meehan et al., 2011b). Yet the effect of pairing excitatory rTMS over *ipsilesional* S1 (IL-S1) with skilled motor practice in individuals with in chronic stroke has yet to be investigated.

The primary objective of the current study was to determine whether 5 Hz rTMS over IL-S1 paired with skilled motor practice would result in improvements in motor learning compared to skilled motor practice paired with sham stimulation in individuals with chronic stroke. In addition, we examined whether 5 Hz rTMS over IL-S1 was associated with persistent increases in cutaneous somatosensation of the hemiparetic hand, and if the stimulation effects would generalize to alter motor function of the hemiparetic arm.

## Materials and methods

### Participants

Fifteen individuals (4 females, mean age: 66.2 years) with first time, chronic stroke (>6 months post) were recruited from the local community (Table 1). Exclusion criteria included: (1) significant cognitive impairment [<20 on the Montreal Cognitive Assessment (MoCA)] (Nasreddine et al., 2005), (2) severe upper extremity impairment [Arm Motor Fugl-Meyer (FM) score of <15 (Fugl-Meyer et al., 1975)], or (3) contraindication to TMS (Rossi et al., 2009). The research ethics board of the University of British Columbia approved all procedures. Informed, written consent was obtained from all participants according to the Declaration of Helsinki.

**Table 1:**
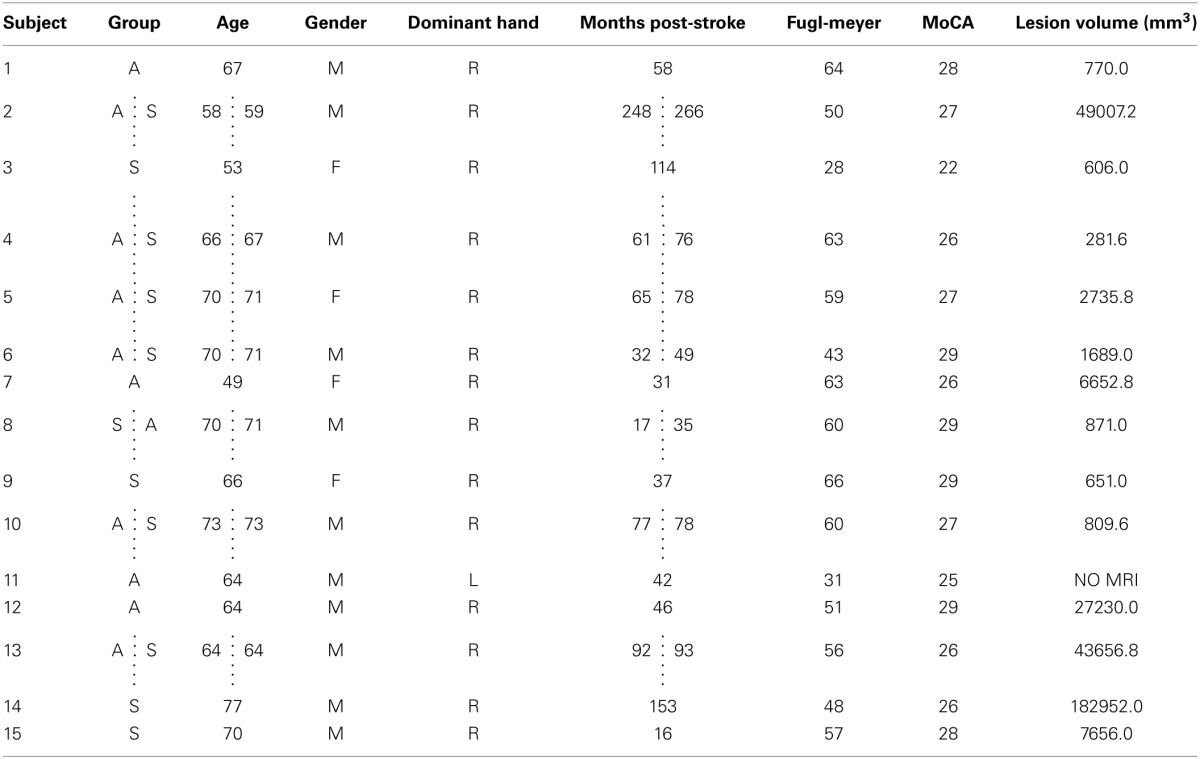
**Participant demographics and lesion location**.

Abbreviations: A, active group; S, sham group. M, male; F, female. R, right; L, left. Divided cells indicate first and second time participating, respectively. Note: No MRI was obtained for subject 11 due to a contraindication.

Participants were pseudo-randomized into either the Active (5 Hz) or Sham rTMS groups, using a custom software to evenly distribute age, gender, and level of physical impairment (Arm Motor FM score) (Fugl-Meyer et al., 1975). Given that FM scores stabilize by 90 days post-stroke (Duncan et al., 1992), we employed this measure to index baseline levels of motor impairment. Each individual was naïve to group assignment. After a minimum washout period of 4 weeks (Fregni et al., 2006; Ackerley et al., 2010), each individual was invited back to participate in the study a second time in the opposite group. However, due to experimental mortality only 7 of the 15 were able to return (6 Active → Sham, 1 Sham → Active), while 8 individuals participated only once (4 Active, 4 Sham). Thus, in total 11 individuals were assigned to each group.

### Procedure

**Figure 1:**
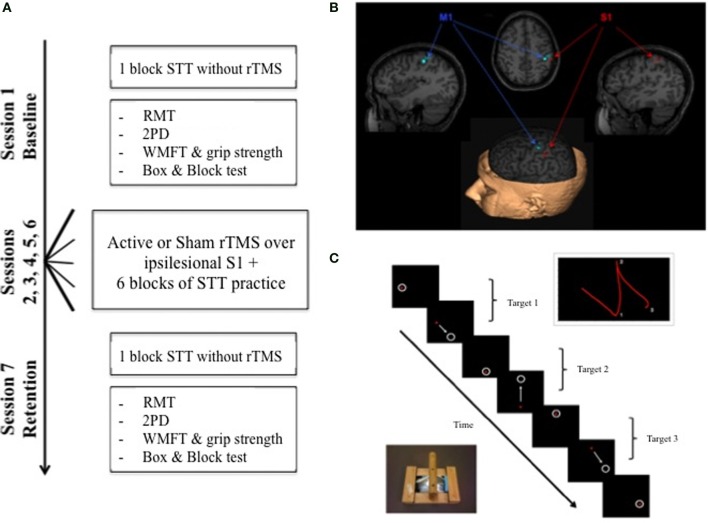
**(A)** Experimental overview. At the baseline session on day 1, STT performance was assessed along with RMT, 2PD, WMFT, and Box & Blocks performance. Five sessions of rTMS paired with STT practice were completed on separate days (days 2–6). A delayed no-rTMS retention test was administered on a separate day 7 to assess motor learning; all baseline measures were re-assessed. **(B)** Example of target locations in BrainSight™ for M1 and S1. **(C)** Schematic of the experimental motor learning task, the STT, showing the adapted mouse, a sample progression of targets and illustration of a path of movements between 2 targets. STT, Serial Tracking Task; RMT, Resting Motor Threshold; 2PD, 2 Point Discrimination; WMFT, Wolf Motor Function Test; rTMS, repetitive Transcranial Magnetic Stimulation.

The experiment was conducted over seven sessions, each separated by no more than 3 days (Figure 1A). On Day 1, one block of a Serial Targeting Task (STT) using the hemiparetic limb, 2-point discrimination (2PD), an abbreviated version of the Wolf-Motor Function Test (WMFT) (Wolf et al., 2001; Bogard et al., 2009), the Box and Block Test of manual dexterity (BBT) (Mathiowetz et al., 1985a), and resting motor threshold (RMT) were assessed. On Days 2–6 participants received rTMS prior to completing a set of six blocks (72 trials/block) of the STT. One group (Active) received 5 Hz rTMS over IL-S1. The other group (Sham) received sham stimulation that looked and sounded like active 5 Hz rTMS but did not induce a current. The interval between stimulation and initiation of the motor practice set was typically less than 5 min.

To assess changes in motor learning, as well as cutaneous somatosensation, motor function, and cortical excitability, a no-rTMS delayed retention test was performed on Day 7. Similar to Day 1, this consisted of one block of the STT, 2-PD, the abbreviated WMFT, the BBT, and RMT assessment.

### Serial targeting task

Motor learning was assessed using a goal-directed, visuomotor task, the Serial Targeting Task (STT) (Figure 1C) (Meehan et al., 2011b). Participants were seated in front of a computer monitor, holding a wireless mouse (Microsoft Wheel Mouse) in a custom frame with their hemiparetic hand. The goal was to move the cursor between sequentially appearing 28 mm diameter targets in one of nine possible locations as quickly and accurately as possible. One target was in the center of the screen, and the other eight formed an equidistant circular array at a 96 mm radius; the tangent distance between the azimuth locations was 75 mm. Only one target was visible at any given time; to initiate the appearance of the next target, participants were required to hold the cursor within the current target for 500 ms. After a 500 ms inter-stimulus interval, the next target appeared. Vision of the hemiparetic hand was blocked to isolate the specific effects of somatosensation from visual feedback on motor performance (Vidoni et al., 2010). Cursor position was sampled at 200 Hz, according to the Cartesian pixel coordinates (Labview v.8.1; National Instruments Co.), and then converted to distance offline by calculating the tangent between each subsequently sampled X, Y pixel coordinate. Pixel distance was converted to mm according to screen resolution (1280 × 1050) and display size (42.25 × 34.65 mm) giving a conversion factor of 3.3 pixels/mm. The resulting magnitude by time waveform was low-pass filtered at 5 Hz.

Each block of STT practice contained 9 alternating repetitions of 8 element sequences (5 random, 4 repeated). Random sequences assessed changes in non-specific motor control, whereas repeated sequences allowed the evaluation of these effects on implicit motor sequence learning (Boyd and Winstein, 2006). The duration of each block was dependent on individual performance, however on average one block took ~4 min to complete. For uniformity of task difficulty, each participant practiced the same set of trials. For the 7 individuals who participated twice, each sequence was reversed; this enabled practice of a novel sequence of equal difficulty and prevented practice from the first part of the crossover to influence performance in the second.

Motor performance was evaluated at baseline, during the 5 rTMS plus practice days, and at retention. Three primary variables were extracted using custom Labview software: (1) Response Time (time from target appearance to the presentation of the next target, corrected for the 500 ms stationary period and the 500 ms inter-target interval), (2) Peak Velocity (maximum velocity reached during the initial ballistic component of the movement), and (3) Cumulative Distance (total distance in mm that the participant's cursor traveled). For each variable, the average of the 8 elements within each sequence was derived. Sequences within each block were then averaged according to type (random or repeated).

### Transcranial magnetic stimulation (TMS)

Prior to the beginning of the experiment, a high resolution anatomical MRI (*TR* = 12.4 ms, *TE* = 5.4 ms, flip angle θ = 8°, FOV = 256 mm, 170 slices, 1 mm thickness) was obtained for each participant, except for one individual with a contraindication to MRI. Image acquisition was conducted at the UBC MRI Research Centre on a Philips Achieva 3.0T whole body MRI scanner (Phillips Healthcare, Andover, MD) using an eight-channel sensitivity encoding head coil (SENSE factor = 2.4) and parallel imaging. MRIcron software (Rorden et al., 2007) was used to trace lesion volumes for each individual, and AFNI software (Cox, 1996) was used to locate the centroid of each stroke lesion (Table 2). The anatomical images were imported into BrainSight™ TMS neuronavigation software (v2.0) for stereotactic guidance during rTMS (Figure 1B). The MNII52 standard brain template was used for the one individual who had no MRI scan. Marking each trajectory in BrainSight™ ensured consistency in the application of stimulation both within and across sessions.

**Table 2:**
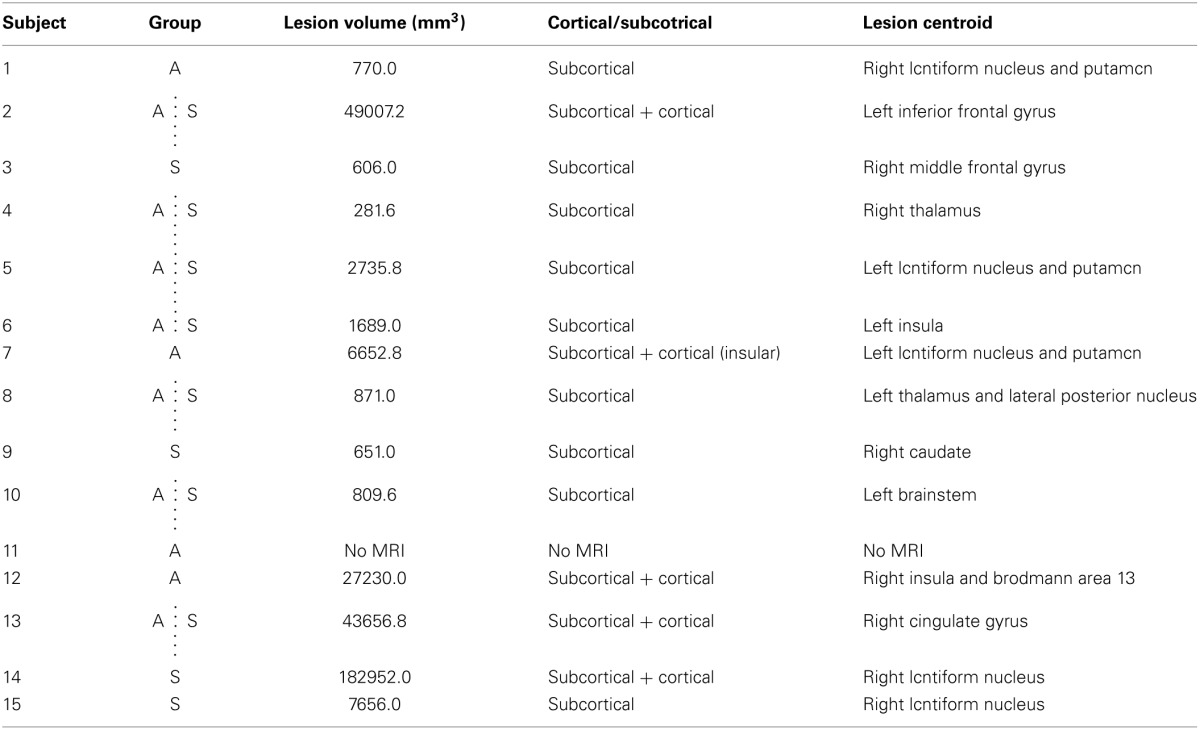
**Participant lesion descriptions**.

Abbreviations: A, active group; S, sham group. Divided cells indicate first and second time participating, respectively. Note: No MRI was obtained for subject 11 due to a contraindication.

**Figure:**
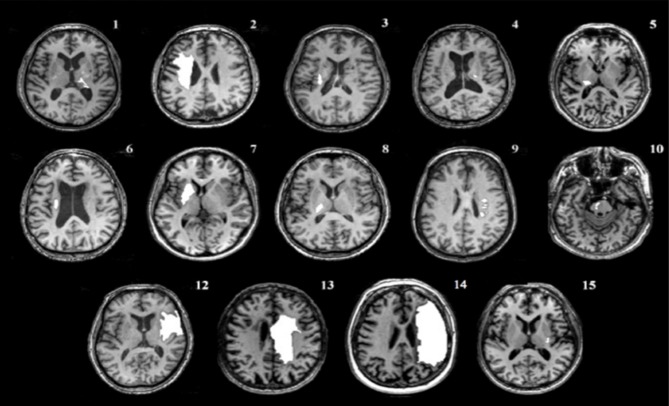


Individuals were seated in a reclined chair and instructed to keep their arms at rest. Surface electromyography (EMG) was recorded from the extensor carpi radialis (ECR) muscles. EMG activity was visually inspected online by two experimenters to ensure that the recording was not contaminated by background muscle activity. Single pulses were applied over the hand knob of M1 with the coil oriented tangentially to the scalp, and the handle at 45° to the midline in a posterior-lateral orientation. Motor evoked potentials (MEPs) were elicited at suprathreshold intensity in order to locate the ECR “hotspot” in M1. RMT was then defined as percent stimulator output to produce a MEP of at least 50 μV peak-to-peak, in five out of ten trials, respectively (Pascual-Leone, 2002).

On Days 2–6, 5 Hz active or sham rTMS was applied over IL-S1 prior to STT practice. A 70-mm figure-of-eight air-cooled coil connected to a Magstim Super Rapid stimulator (Magstim Company, Ltd., Wales, UK) was used to deliver biphasic stimulation that produces a current flow in a posterior-anterior, then anterior-posterior direction, with a pulse width of 400 *us*. The TMS coil delivers stimulation to a relatively focal point of the cortex (Cohen et al., 1990; Pascual-Leone, 2002) specific enough to target specific areas of the cortex independently (Wassermann et al., 1996; Chouinard et al., 2005). S1 stimulation was delivered ~2 cm posterior to the M1 ECR “hotspot” (Maldjian et al., 1999; Tegenthoff et al., 2005), directly over the crown of the post-central gyrus using the T_1_ scan to guide placement (Vidoni et al., 2010; Meehan et al., 2011b, 2013). Prior to administering rTMS, single suprathreshold pulses, at ~110% of RMT were used to verify isolation of S1 from M1, as evidenced by a lack of MEP when S1 was stimulated above RMT (Eshel et al., 2010).

The active rTMS protocol consisted of 24 trains of 5 Hz pulses at an intensity of 90% RMT for 10 s, with 5 s rest in between (1200 pulses in total). This stimulation protocol was selected based on our past work considering the effect of stimulation over the dorsal premotor cortex (Boyd and Linsdell, 2009; Meehan et al., 2013), and falls within safely defined rTMS limits (Rossi et al., 2009). The Sham group underwent the identical procedure using a custom sham coil (Magstim Company, Ltd., Wales, UK).

### Cutaneous somatosensation

Static, tactile 2PD was used to assess changes in cutaneous somatosensation in the hemiparetic hand (van Nes et al., 2008). During testing, participants were at rest with forearms supinated and vision obscured. The two arms of an aesthesiometer (Baseline® Aesthesiometer) were simultaneously placed on the thenar eminence with enough force to depress the skin for 1 s; participants reported whether they felt one or two contacts. Sequential adjustments of the arms were made in 2 mm increments to a maximum of 30 mm. Sensory threshold was the distance where participants correctly reported feeling two points of contact 7 of 10 times (van Nes et al., 2008). Catch trials were randomly applied where only one arm of the aesthesiometer was used. One individual who participated in both groups had severe sensory loss in the hemiparetic thumb due to a previous mechanical hand injury (partial thumb amputation) prior to stroke, and was excluded from 2PD analysis. In addition, 2PD was not collected for two members of the Active group owing to clerical error, therefore group sizes for 2PD analysis were *n* = 8 (Active) and *n* = 10 (Sham).

### Motor function

Three task-performance items were selected from the original version of the WMFT (Wolf et al., 2001) to briefly assess affected upper extremity motor function: time to pick up can, pick up paperclip, and fold towel. Raw movement times for each task were calculated as a projected task rate per minute of task performance, to ensure normality (Hodics et al., 2012). In addition, grip strength (Mathiowetz et al., 1985b) of the affected hand was assessed using a Jamar ® Hand Dynamometer (5030J1). An average of the three attempts was calculated. Finally, the BBT was used to measure unilateral manual dexterity of the affected hand (Mathiowetz et al., 1985a). BBT score was the number of blocks transferred in 1 min.

### Data analysis

All analyses were performed with SPSS (v20) software. Group demographics were compared using independent samples *t*-tests. Descriptive and Shapiro-Wilk statistics were used to evaluate normality. Non-parametric tests were used for assessing group differences in baseline 2PD thresholds due to unequal sample sizes.

To compare the effects of active- to sham-rTMS interventions, univariate linear mixed effects models were constructed. This statistical model design has the benefit of accounting for subject effects in the partial crossover design employed and for missing data. Dependent measures of tracking performance (Response Time, Peak Velocity and Cumulative Distance), 2PD threshold, WMFT task performance rate, grip strength, BBT score, and RMT were assessed. Group, Day, and Sequence (for STT) were considered as fixed effects in the model. A Subject term was included in the random effects model. A variance components covariance structure was specified and an intercept term was included in the random effects model. Significance level was set at *p* < 0.05 for Type III *F*-tests of the fixed and interaction effects in the model.

## Results

### Baseline group characteristics

The Active and Sham groups did not differ significantly in mean age, time post-stroke, FM score, or lesion volume (Tables 1, 2) (all *p* > 0.4). There were no significant group differences in baseline STT performance for any measure (*p* > 0.7).

### Primary outcome measure

#### Motor learning

**Figure 2:**
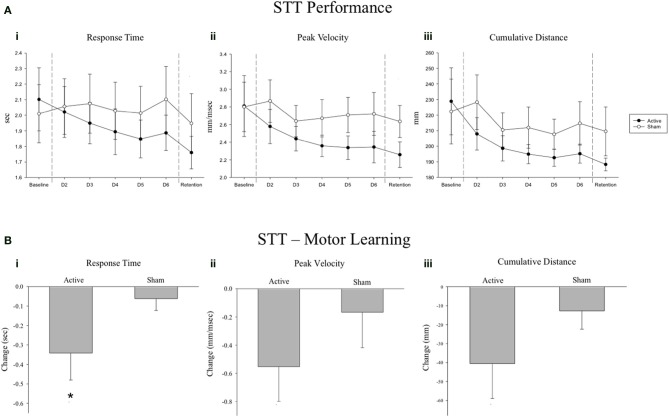
**(A)** Serial tracking task (STT) mean performance values across all 7 days of the experiment for the Active and Sham groups. A significant Group * Day interaction was observed for (i) Response Time, (ii) Peak Velocity and (iii) Cumulative Distance tracked (*p* ≤ 0.044). **(B)** Change scores from baseline to retention for the Active and Sham groups. Negative change scores reflect performance improvements from baseline to retention, as reflected by reduced response times, lower peak velocities and less cumulative distance traveled, respectively. A significant Group * Day interaction was observed for Response Time (i; ^*^p = 0.011), but not for Peak Velocity (ii; *p* = 0.122) or Cumulative Distance tracked (iii; *p* = 0.081). Error bars are s.e.m.

We examined the effect of 5 Hz rTMS over IL-S1 paired with skilled motor practice of the STT on motor performance across the 7 days of the experiment for both groups (Figure 2A). There was no significant Group × Sequence × Day interaction observed for any of the three primary variables (*p* > 0.7). A significant Group × Day interaction was found for Response Time [*F*_(1, 286.04)_ = 13.016, *p* = 0.0004], Peak Velocity [*F*_(1, 285.95)_ = 4.111, *p* = 0.044], and Cumulative Distance [*F*_(1, 285.92)_ = 4.076, *p* = 0.044]. To assess group-specific motor learning related changes (Active vs. Sham), we selectively evaluated mean performance values from the no-rTMS baseline and the no-rTMS retention tests and did not include performance on the rTMS + practice days (Figure 2B). A significant Group × Day interaction was observed for Response Time [*F*_(1, 66.05)_ = 6.761, *p* = 0.011], but not Peak Velocity [*F*_(1, 65.87)_ = 2.456, *p* = 0.122] or Cumulative Distance [*F*_(1, 65.54)_ = 3.134, *p* = 0.081]. A significant main effect of Day was observed for Response Time [*F*_(1, 66.05)_ = 14.786, *p* = 0.0003], Peak Velocity [*F*_(1, 65.87)_ = 8.645, *p* = 0.005] and Cumulative Distance [*F*_(1, 65.54)_ = 13.341, *p* = 0.001], suggesting that STT practice benefitted both groups.

### Secondary outcome measures

#### Cutaneous somatosensation

**Figure 3:**
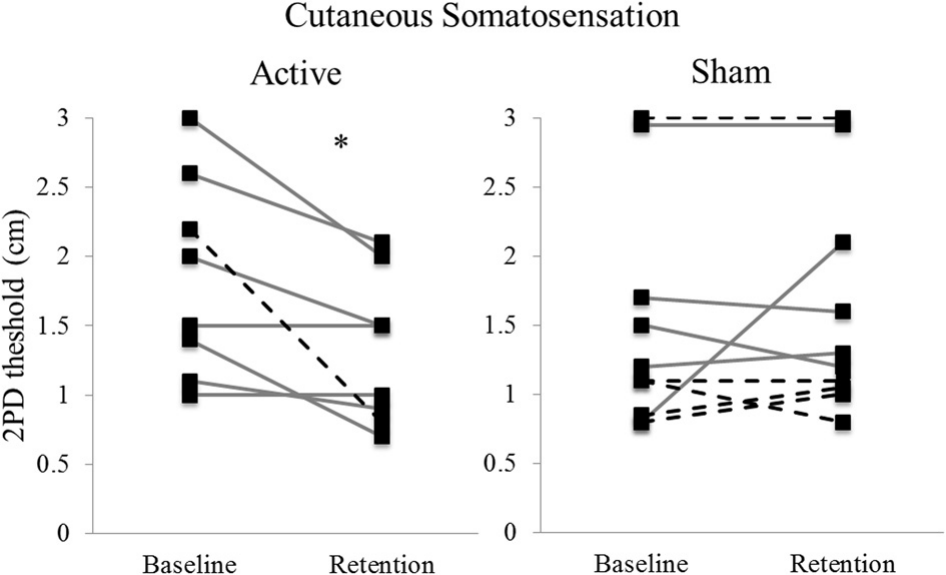
**Individual thresholds for 2-point discrimination at baseline and retention, by stimulation type**. Lower values indicate better somatosensory discrimination (i.e., less distance between stimulation points). Solid lines indicate first time participation, dashed lines indicate second time (crossed over) participation. (*n* = 8 Active; 10 Sham). ^*^p = 0.007.

Five Hz rTMS over IL-S1 paired with motor practice improved 2PD threshold of the hemiparetic hand (group median: 2.00 cm at baseline, 1.25 cm at retention) compared to sham stimulation paired with practice (group median: 1.15 cm at baseline, 1.25 cm at retention), as indicated by a significant Group × Day interaction [*F*_(1, 21.15)_ = 8.793, *p* = 0.007; Figure 3]. The two groups did not differ significantly at baseline (*p* = 0.27).

#### Motor function

No significant Group × Day interaction was found for pick up can rate (*p* = 0.71), pick up paperclip rate (*p* = 0.59), or fold towel rate (*p* = 0.72). In addition, no significant Group × Day interaction was detected for dynamometer grip strength (*p* = 0.96) or BBT score (*p* = 0.93).

#### Motor cortex excitability

To determine whether the changes observed with 5 Hz rTMS over IL-S1 may be attributed to altered ipsilesional M1 cortical excitability, we also evaluated RMT at baseline and retention. No significant Group × Day interaction was observed (*p* = 0.07; Supplementary Figure 1).

## Discussion

We demonstrated that 5 Hz rTMS over IL-S1 paired with skilled motor practice enhanced motor performance and learning of a novel skilled motor task in individuals with chronic stroke. The benefits of 5 Hz rTMS over IL-S1 paired with motor practice were also associated with significant improvements in cutaneous somatosensation, as measured by 2PD. However, a significant effect was not observed for measures of motor function (abbreviated WMFT) or manual dexterity (BBT).

### STT performance as an index of motor learning

Our primary outcome measure was change in STT performance across 5 days practice and at a delayed, no-rTMS retention test. Over the course of the experiment, greater improvements in motor performance were observed in the Active group across reaction time, peak velocity, and cumulative distance moved (Figure 2A). Despite the observation of larger changes from baseline to retention in the Active group for all three variables (Figure 2B), when only baseline and retention data were considered, the group by day interaction failed to reach statistical significance for peak velocity and cumulative distance moved. This can likely be attributed to our small sample size and a lack of statistical power, as the number of data points was drastically reduced when the 5 days of practice data were excluded from the analysis. Nevertheless, a significant reduction in response time in the Active group at retention suggests that motor learning was indeed enhanced by 5 Hz rTMS over IL-S1. This effect was noted regardless of sequence type (random or repeated). In other words, active stimulation over IL-S1 did not yield sequence-specific benefits, but rather led to a generalized improvement of motor performance that was evident in both repeated and random sequence tracking. This is consistent with our past work demonstrating a reduction in non-specific motor control after inhibitory 1 Hz rTMS over S1 in healthy adults (Vidoni et al., 2010), as well as improved generalized motor learning following cTBS over CL-S1 in individuals with chronic stroke (Meehan et al., 2011b). The improvement in response time was most pronounced at the no-rTMS retention test, suggesting that 5 Hz rTMS over IL-S1 paired with STT practice influenced motor learning by facilitating offline motor memory consolidation mechanisms (Robertson et al., 2004; Boyd and Linsdell, 2009; Wilkinson et al., 2010; Dayan and Cohen, 2011).

Interestingly, in both groups the reduction in total response time and cumulative distance traveled occurred at the expense of peak velocity, which also decreased with repeated practice of the STT. This pattern suggests that participants developed improved motor control by taking more direct, guided trajectories between the starting point and end target. This was more pronounced for individuals who received 5 Hz rTMS over IL-S1, which was intended to increase cortical excitability of S1. In contrast, individuals who received sham stimulation prior to practice did not show the same magnitude of behavioral change. Improvements in perceptual learning following 5 Hz rTMS over the sensory cortex have been documented before (Ragert et al., 2003), albeit with a slightly different rTMS protocol. To our knowledge the current study is the first to show improved motor learning associated with active 5 Hz rTMS over IL-S1 paired with motor practice in a chronic stroke population.

### Cutaneous somatosensation, motor function, and motor cortex excitability

Five days of 5 Hz rTMS over IL-S1 paired with STT practice also improved cutaneous somatosensation of the hemiparetic hand, as measured by 2PD. This observation corresponds with previous findings that a modified 5 Hz rTMS protocol applied over the finger area of S1 decreases 2PD thresholds and enlarges the corresponding cortical representation in healthy individuals (Tegenthoff et al., 2005). Nevertheless, the improved somatosensory discrimination observed here demonstrated limited transfer to the abbreviated WMFT or the BBT. This is in contrast to our past work demonstrating that cTBS over CL-S1 paired with the same STT induced improvements not only in motor learning but also in the WMFT (Meehan et al., 2011b). It is possible that stimulation over the contralesional side imparts effects across a larger portion of the sensorimotor network, given the extensive transcallosal connections between the hemispheres (Fling et al., 2013), leading to broader functional gains. However, given the preliminary nature of the current study and that of Meehan et al. (2011b), the underlying neural mechanisms remain unclear. Further work is necessary to determine the optimal site(s), and protocols of stimulation to promote transfer to functionally relevant domains.

In addition to examining changes in motor behavior and somatosensation, we assessed the excitability of the adjacent motor cortex at baseline and retention. Despite the observed increase in motor performance from baseline to retention with 5 Hz rTMS over IL-S1 plus motor practice, a single pulse TMS measure of ipsilesional corticospinal excitability (RMT) showed no significant changes in either group. It is possible that, given our small sample size, we were underpowered to detect subtle changes in M1 excitability. However, an alternative explanation may be that increased IL-S1 excitability following 5 Hz rTMS paired with motor practice resulted in increased functional connectivity between IL-S1 and IL-M1 during sensory-guided movement. Indeed, this is in line with current theories of motor learning suggesting that skilled behavior arises from a complex interaction between sensory and motor systems and gives rise to an internal model for movement (Ito, 2000).

### Study limitations

Given the relatively small sample size and pseudo-crossover design, the current work should be interpreted as a preliminary report. Our groups were matched on a number of characteristics, however there was a broad range of lesion locations. While this heterogeneity is representative of a clinical stroke population, and fMRI studies have shown that cortical and subcortical strokes behave similarly in terms of post-stroke hemispheric activation imbalances in M1 and S1 (Calautti et al., 2007), there is evidence to suggest that measures of cortical excitability may manifest differently according to the cortical/sub-cortical nature of the infarct (Shimizu et al., 2002). Having 7 of 15 participants cross over between groups had the benefit of reducing some between-group variability, and potential crossover effects were taken into account statistically using mixed effects modeling. Nevertheless, the possibility for carry-over effects or bias due to our small sample cannot be entirely ruled out. Finally, another limitation of the current study is that we did not directly assess excitability changes in S1. While we did measure cutaneous somatosensation, this is an indirect assessment of S1. Further work is needed to better understand the neurophysiological mechanisms involved.

## Conclusion

The current findings suggest that 5 Hz rTMS over IL-S1 paired with skilled motor practice may enhance motor learning in individuals with chronic stroke. This enhancement is concurrent with improvements in cutaneous somatosensation. Taken together with past work, these results reinforce the importance of sensory cortex activity in motor skill learning, and suggest that rTMS-based activity modulation may be effective in enhancing motor learning during post-stroke rehabilitation.

### Conflict of interest statement

The authors declare that the research was conducted in the absence of any commercial or financial relationships that could be construed as a potential conflict of interest.

## Supplementary material

Supplementary Figure 1 **Individual resting motor threshold (RMT) values at baseline and retention by stimulation type**. Values in the first column represent subject number. Values in the second and third columns represent percent stimulator output. (*n* = 11/group).

## Abbreviations

rTMS: repetitive transcranial magnetic stimulation
M1: primary motor cortex
S1: primary sensory cortex
cTBS: continuous theta-burst stimulation
CL: contralesional
IL: ipsilesional
MoCA: Montreal Cognitive Assessment
FM: Fugl Meyer score
STT: serial targeting task
2PD: 2 point discrimination
WMFT: Wolf Motor Function Test
BBT: box and blocks test
RMT: resting motor threshold
EMG: electromyography
MEP: motor evoked potential
ECR: extensor carpi radialis.
