# Supplementary figures and images for: 5 Hz repetitive transcranial magnetic stimulation over the ipsilesional sensory cortex enhances motor learning after stroke
Source: Front Hum Neurosci. 2014 Mar 21;8:143. doi: 10.3389/fnhum.2014.00143 (PMC3968757; doi:10.3389/fnhum.2014.00143)

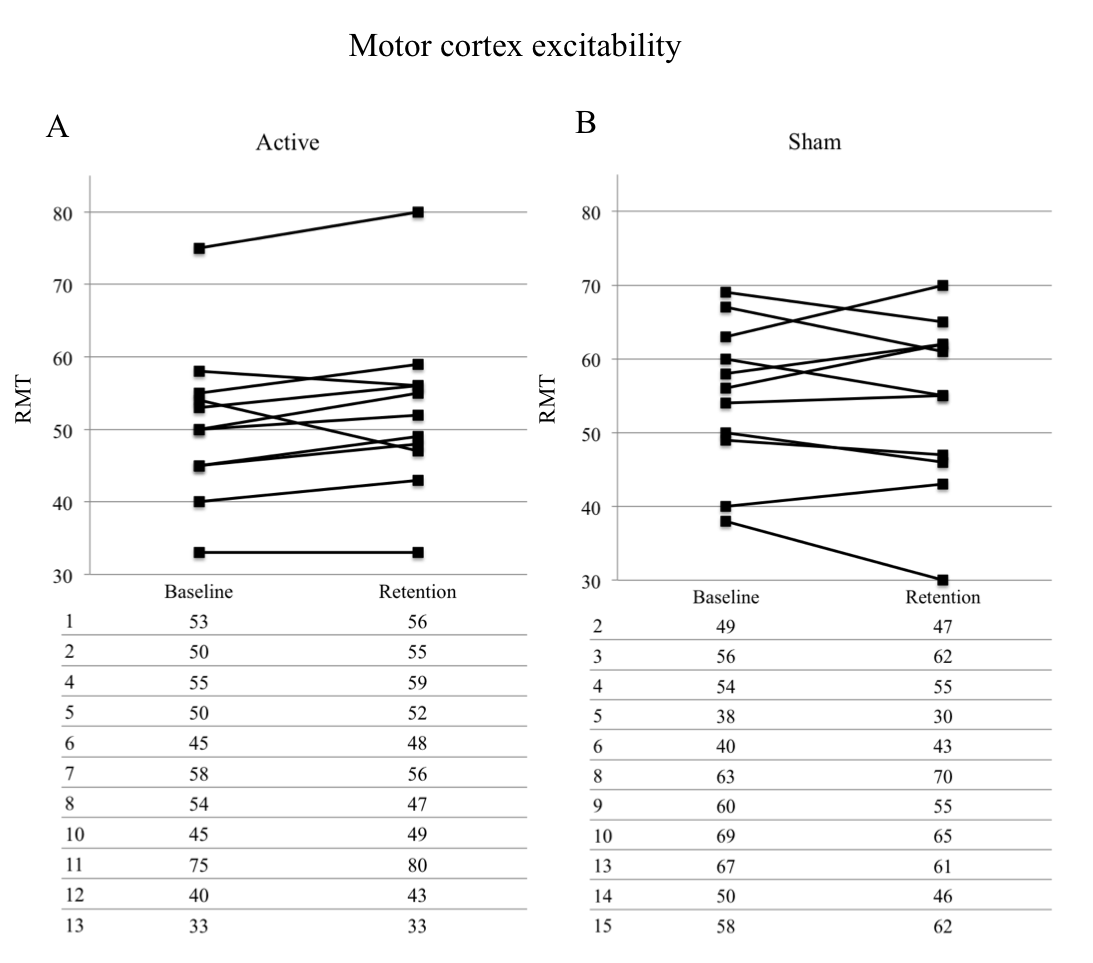

Supplement: Supplementary Figure 1 — Individual resting motor threshold (RMT) values at baseline and retention by stimulation type. Values in the first column represent subject number. Values in the second and third columns represent percent stimulator output. (n = 11/group). [file Figure1.TIFF]
